# Advancing Hydrogel-Based 3D Cell Culture Systems: Histological Image Analysis and AI-Driven Filament Characterization

**DOI:** 10.3390/biomedicines13010208

**Published:** 2025-01-15

**Authors:** Lucio Assis Araujo Neto, Alessandra Maia Freire, Luciano Paulino Silva

**Affiliations:** 1 Embrapa Genetic Resources and Biotechnology, Laboratory of Nanobiotechnology (LNANO), Brasília 70770-917, DF, Brazil; lucioaraujo@ufpr.br (L.A.A.N.); alessandra.maia.freire@gmail.com (A.M.F.); 2 Postgraduate Program in Pharmaceutical Sciences, Federal University of Paraná (UFPR), Curitiba 80210-170, PR, Brazil; 3 Postgraduate Program in Nanoscience and Nanobiotechnology, University of Brasilia (UnB), Brasília 70910-900, DF, Brazil

**Keywords:** Teachable Machine, machine learning, artificial intelligence, confusion matrix, hydrogel

## Abstract

**Background:** Machine learning is used to analyze images by training algorithms on data to recognize patterns and identify objects, with applications in various fields, such as medicine, security, and automation. Meanwhile, histological cross-sections, whether longitudinal or transverse, expose layers of tissues or tissue mimetics, which provide crucial information for microscopic analysis. **Objectives**: This study aimed to employ the Google platform “Teachable Machine” to apply artificial intelligence (AI) in the interpretation of histological cross-section images of hydrogel filaments. **Methods**: The production of 3D hydrogel filaments involved different combinations of sodium alginate and gelatin polymers, as well as a cross-linking agent, and subsequent stretching until rupture using an extensometer. Cross-sections of stretched and unstretched filaments were created and stained with hematoxylin and eosin. Using the Teachable Machine platform, images were grouped and trained for subsequent prediction. **Results**: Over six hundred histological cross-section images were obtained and stored in a virtual database. Each hydrogel combination exhibited variations in coloration, and some morphological structures remained consistent. The AI efficiently identified and differentiated images of stretched and unstretched filaments. However, some confusion arose when distinguishing among variations in hydrogel combinations. **Conclusions**: Therefore, the image prediction tool for biopolymeric hydrogel histological cross-sections using Teachable Machine proved to be an efficient strategy for distinguishing stretched from unstretched filaments.

## 1. Introduction

Artificial intelligence (AI) is a field of computer science focused on the development of systems and algorithms capable of emulating human intelligence to perform tasks such as problem-solving, decision-making, and pattern recognition [1]. One of the significant applications of AI is in image analysis, also known as image recognition or computer vision. This involves the utilization of machine learning techniques, particularly deep neural networks, to analyze and interpret visual data, enabling computers to comprehend, classify, and make predictions based on images or videos [2,3].

The analysis of images with AI technology employs sophisticated algorithms to extract intricate patterns and features from images, enabling the accurate classification and differentiation of objects, structures, or characteristics within the images [4]. This technology finds applications in areas such as medical diagnosis, quality control, and autonomous systems [5]. An example of a tool that allows users to train AI to recognize patterns in images is the “Teachable Machine”, a tool developed by Google’s Creative Lab and available free of charge. This tool empowers individuals to train their own AI models without extensive coding knowledge, enabling machine learning-based AI to differentiate objects based on user-provided examples. This process contributes to democratizing and popularizing AI resources for a broader range of users [6,7]. AI-powered image analysis, which extracts patterns and features to classify objects, has applications in areas such as medical diagnosis, quality control, and autonomous systems and has been popularized by tools such as Teachable Machine, which allows AI training without advanced programming knowledge [8].

The use of AI in image prediction has found widespread applications across various sectors. In the healthcare field, AI algorithms can assist medical professionals in disease diagnosis by analyzing medical images such as X-rays [9], MRI scans [10], and CT scans [11]. Furthermore, AI image prediction has been extensively utilized in industries such as manufacturing for quality control, agriculture for crop monitoring, and e-commerce for product recommendation systems. These applications rely on AI to analyze extensive datasets of images and provide valuable insights or predictions [12]. Overall, AI has revolutionized image prediction, enabling computers to comprehend visual data, and its applications span multiple sectors, enhancing efficiency, accuracy, and decision-making processes [13].

Histological images provide detailed visual representations of tissues at the cellular level, essential for understanding tissue structure and pathological conditions [14]. Similarly, histological images from hydrogels provide valuable insights into the structural characteristics of hydrogel materials, aiding in their analysis and applications in various fields, including biomedicine and materials science [15]. The techniques for staining these samples involve the application of specialized dyes to tissue samples, enabling the visualization and differentiation of specific cellular structures and components for pathological analysis, such as hematoxylin and eosin. These techniques are essential for enhancing morphological details and facilitating pathological identification in tissue samples [16,17].

Therefore, to explore a new application of AI for image interpretation, the present study aims to utilize the Google platform “Teachable Machine” for the learning and prediction of histological cross-section images of hydrogel filaments, distinguishing those mechanically stretched from those with distinct morphological characteristics. Hydrogels, like the ones studied here, have significant potential for applications in three-dimensional (3D) cell culture systems. Due to their biocompatibility, tunable mechanical properties, and ability to mimic the extracellular matrix, these materials can support cellular adhesion, proliferation, and differentiation. Such characteristics make them promising candidates for biomedical research, including the development of engineered tissues like nerves, tendons, and muscles.

## 2. Materials and Methods

### 2.1. Experimental Design, Filament Production from Hydrogels, and Electromechanical Tension

For the preparation of biopolymeric hydrogels, the concentrations of sodium alginate, gelatin, and calcium chloride were determined using the Chemoface^®^ software (version 1.65) with a central composite experimental design. Table 1 shows the seven selected combinations generated by the software. As a solubilizing agent, Dulbecco’s Modified Eagle Medium (DMEM) cell culture medium was chosen. Therefore, each combination was identified by adding the word DMEM followed by the generated combination number.

The hydrogels were prepared from combinations of the biopolymers sodium alginate (CRQ, Brazil) and gelatin (Vetec, Brazil) following the protocol defined by the experimental design. At room temperature, a glass beaker (Pyrex^®^, USA) was placed on a magnetic stirring plate (Lucadema, Brazil), and sodium alginate was added for solubilization in each solvent, followed by heating. Gelatin was added to the same beaker, and the temperature was gradually increased to approximately 40 °C for 30 min until complete solubilization and homogeneity were achieved. Subsequently, the hydrogels were stored at room temperature in 5 mL disposable luer-lock syringes (Rynco, Brazil) and manually extruded into glass containers containing a calcium chloride solution (Dinâmica, Brazil) as the crosslinking agent, following the wet spinning technique. A 14-gauge needle was used for extrusion. These filaments were immersed for approximately 10–15 min, washed with PBS, and then gently dried on paper towels.

Finally, using an electromechanical extensometer attached to a load cell (MTS Insight^®^, USA), the produced filaments were clamped at their ends and tested in triplicate until rupture. The samples were subjected to stress using TestWorks^®^ 4.10 software at a speed of 10 mm/min.

### 2.2. Light Microscopy and Histology of Hydrogel Filaments

The filaments produced using hydrogels based on the combinations of selected biopolymers were processed for evaluation under light microscopy. For this purpose, extruded filaments were used both before and after being stretched to their rupture point by an electromechanical extensometer. Thus, the analyzed biomaterials were divided into two groups: unstretched filaments (F) and stretched filaments (FE). Approximately 1 cm fragments of each filament were placed separately in 15 mL Falcon-type tubes and identified.

To begin the processing, the biomaterials were placed in methanol-Carnoy fixative solution, which consists of 60% methanol (J.T. Backer, USA), 30% chloroform (CRQ, Brazil), and 10% acetic acid (Merck, Germany), for 2 h at room temperature. After removing the fixative, the samples underwent successive steps of decreasing ethanol concentrations (100%, 90%, and 80%) with a 40 min interval between each bath and were kept in 70% ethanol overnight at 4 °C. After this period, the samples were dehydrated using increasing ethanol concentrations (80%, 90%, and 100% twice), with a 40 min interval between each bath. Subsequently, the clearing process took place, during which the samples were kept in ethanol/xylene solutions in a 1:1 ratio, followed by xylene 1 and then xylene 2, with a 40 min interval in each bath. For embedding the filaments, infiltration with Paraplast (Sigma Life Science, Switzerland) was performed through two 1 h baths (Paraplast 1 and Paraplast 2). After infiltration, the material was embedded in Paraplast blocks, allowed to solidify, and stored at room temperature.

Using the Vibratome 3000 plus equipment (The Vibratome Company, USA), the blocks were sectioned into approximately 5 μm thickness, and the sections were deposited on glass slides, separating longitudinal and transverse cuts; then placed on a heated plate at approximately 40 °C to initiate the drying process; and then taken to an oven at 40 °C overnight. The slides underwent a staining process with hematoxylin and eosin (H&E). The analysis of the micrometric sections of the filaments was conducted using a light microscope (Nikon, Japan) coupled with a digital camera (Digilab, Brazil) and digitally documented using ImageView software version 3.7.

### 2.3. Utilization of Artificial Intelligence as a Strategy for the Classification and Validation of Biomaterial Images

**Figure 1:**
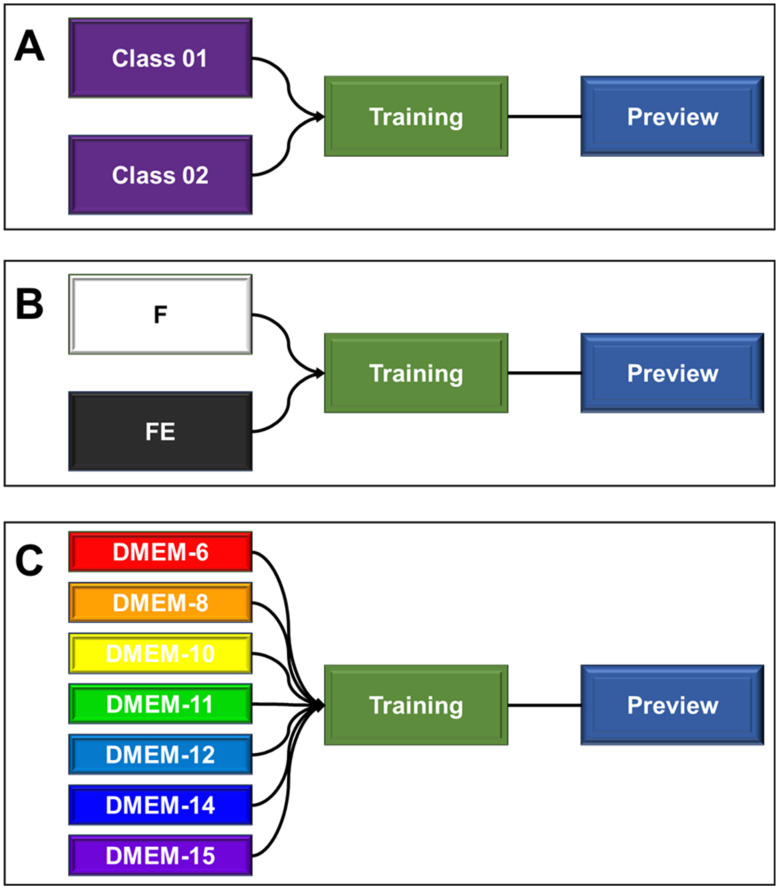
Schematic representation of the teachable machine tool used as a machine learning-based artificial intelligence for predicting image classifications of filaments. (**A**) Initial model structure showing the positions of each structure and its specific function. (**B**) Encoding used for the training and prediction of images of stretched (FE) and unstretched (F) filament sections. (**C**) Encoding used for the training and prediction of images of sections for the seven distinct groups of hydrogel filaments produced.

The light microscopy images of the sections obtained in the previous assay were subjected to a machine learning test, followed by prediction using the Teachable Machine 2.0 AI tool. Figure 1 details the components of the tool used. In this case, the machine learning used for classification relies on the use of categorical data (classes), where the provision of images (“inputs”) and their respective identifications are required for recognition. Subsequently, through the algorithm itself, training occurs and the parameters that relate the images to their corresponding classes are learned. Then, the predicted model is ready to be validated. For this step, it is important to use a new set of images that were not implemented in the previous training but may or may not be associated with any of the trained classes. From this, the tested images will be identified and classified with a confidence percentage (certainty) with respect to the classifications (classes) to which the AI was trained (Figure 1A).

For the training shown in Figure 1B, the classes were identified as “F” (unstretched filaments) and “FE” (stretched filaments). Each class was composed of 250 distinct images (captured with the 4× and 10× microscope objectives). After training, forty-seven images, which were not used in the construction of this trained image dataset, were used for prediction. The hypothesis under evaluation was whether the AI could identify and classify the images into two groups based on the morphologies of stretched and unstretched filaments.

For the training depicted in Figure 1C, the classes were identified as DMEM-6, DMEM-8, DMEM-10, DMEM-11, DMEM-12, DMEM-14, and DMEM-15. To challenge and identify the potential “sensitivity” of prediction, the seven classes were provided with approximately seventy images (stretched and unstretched filaments) each. After training, forty-nine images that were not used in the construction of this trained image dataset were tested for prediction. The hypothesis under evaluation was whether the AI could identify and classify the images based on the seven distinct groups, determining which one they most resemble, guided by their morphologies.

During the AI prediction, it was set that the default threshold for image identification would be above 50% for recognition. After all predictions were made, the resulting values were compiled into a confusion matrix, which is a performance measure that compares the obtained classification results with the ones predicted by the machine, displaying the distribution of records in terms of their actual and predicted classes. The matrix was created using the ChatGPT platform, and the programming was conducted in Python language. All acquired images used are available at this link: https://drive.google.com/drive/folders/12R4aE0TJ4H1GTm9mAzjojqtdvGDNT1Cj?usp=sharing (accessed on 12 December 2024).

## 3. Results and Discussion

### 3.1. Analysis of the Sections Obtained from Each Filament by Light Microscopy

The biopolymeric hydrogel filaments developed in this study were designed with a focus on achieving structural integrity and versatility for diverse applications. By combining sodium alginate and gelatin, cross-linked with calcium chloride, these filaments exhibit tunable mechanical and morphological properties. Such characteristics are crucial for their evaluation under different conditions, including mechanical stretching, to explore their potential use in biomedical applications, such as scaffolds for tissue engineering and 3D cell culture systems. This approach ensures that the materials can provide a robust platform for further investigation into their functionality and adaptability.

**Figure 2:**
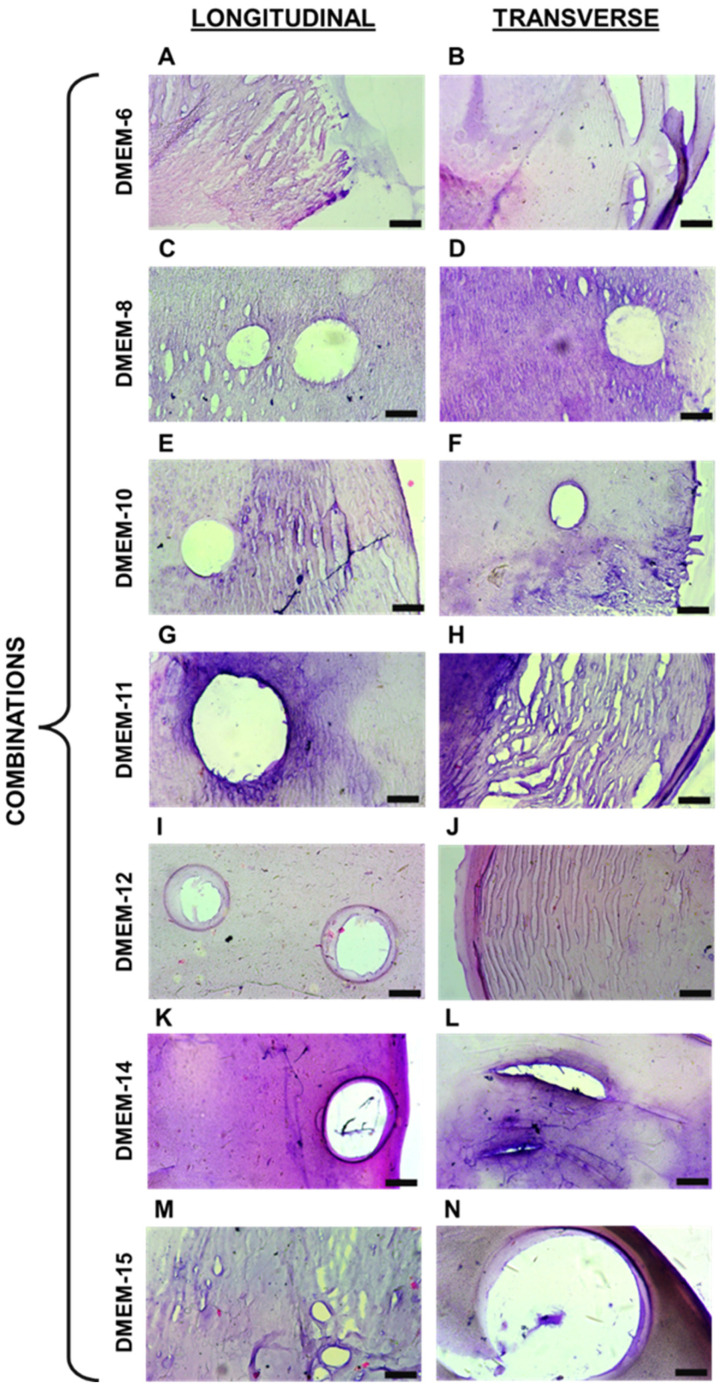
Histological sections of unstretched hydrogel filaments: (**A**,**C**,**E**,**G**,**I**,**K**,**M**) representative images of longitudinal sections; (**B**,**D**,**F**,**H**,**J**,**L**,**N**) representative images of transverse sections. The black mark in the bottom right corner of each image represents 100 μm.

The composition and structural properties of these hydrogels were designed with versatility in mind. Their ability to sustain mechanical stress while maintaining structural integrity could be advantageous for supporting cell growth and tissue formation under dynamic conditions, such as those required for the regeneration of muscular or connective tissues. Regarding the captured images from histological sections, it was initially noticeable that variations in the proportion of biopolymers composing different hydrogels influenced the staining of each material. The dyes used have distinct charges, with hematoxylin having a positive charge, staining alginate [18,19], and eosin having a negative charge, primarily staining proteins like gelatin [20]. It can be inferred that this factor accounts for the variations in the staining of the acquired images. Comparing the images in Figure 2 andFigure 3, it is possible to observe the presence of certain recurring structures in all the samples. For instance, there are numerous pores of various sizes, primarily visible through the 10× objective lens. In addition to these, the biopolymeric mesh can be seen to differ when comparing the stretched and unstretched filaments. For these types of biomaterials, particularly those based on alginate, the presence of pores is quite common, as observed [21], and also associated with other polymers like gelatin [22,23], polyethylene glycol [24], and chitosan [25]. Another factor that may directly contribute to the behavior of these meshes is the concentration of the crosslinking agent used in each combination. Therefore, higher levels of calcium ions can produce distinct effects in the resulting hydrogel network, as previously shown by other authors [26]. These elevated calcium ion concentrations can accelerate crosslinking, affecting handling time and potentially altering mechanical properties intrinsically [27]. However, this denser network can also lead to reduced swelling capacity and altered diffusion characteristics, as observed in other studies [28].

The observed differences in the structural and morphological characteristics of the hydrogel filaments, influenced by variations in biopolymer proportions and cross-linking density, suggest their potential for customizing scaffolds for specific 3D cell culture applications. For instance, hydrogels with higher porosity and tunable stiffness could be tailored for the cultivation of nerve or tendon cells, while more compact and elastic structures might be suitable for muscle tissue engineering.

### 3.2. AI-Based Class Prediction After Learning from Light Microscopy Filament Section Images

**Figure 4:**
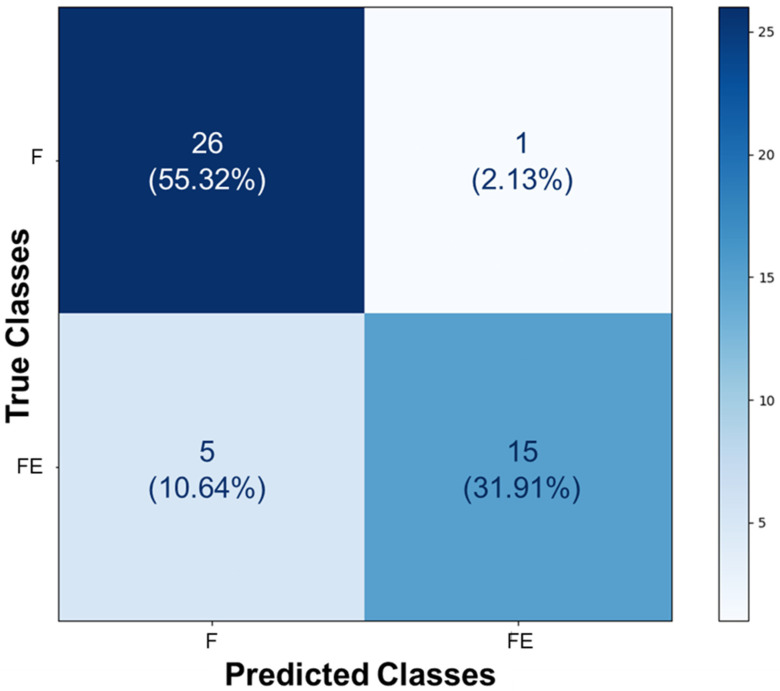
Confusion matrix of the prediction for stretched and unstretched filaments. This matrix presents the proportions corresponding to the identifications of the 47 images, based on the class assignments of F and FE. In total, 26 images belonging to class F (55.32%) were correctly identified as such, and 15 images from class FE (31.91%) were correctly identified as belonging to that class. Five images from class FE (10.64%) were misclassified as F, and only one image from class F (2.13%) was misclassified as FE.

During the process of image classification and prediction using the Teachable Machine tool, the results from the test of identifying stretched and unstretched filaments were collected and added to the confusion matrix. In the section where “True Classes” is written, it signifies the actual value of each image presented to the AI after its training. Conversely, where “Predicted Classes” is written, it represents the value assigned to each image by the AI after prediction. When comparing the obtained results with the actual values of each section, the confusion matrix presented in Figure 4 was constructed. It indicates that twenty-six images belonging to class F (55.32%) were correctly identified as such, and fifteen images from class FE (31.91%) were correctly identified as belonging to that class. Five images from class FE (10.64%) were misclassified as F, and only one image from class F (2.13%) was misclassified as FE. This demonstrates that, even though these images exhibit some colorimetric similarities and morphological differences discussed and highlighted in the previous section, the AI displayed sensitivity in distinguishing the classes as learned.

The ability of AI tools to classify and predict hydrogel characteristics could facilitate the selection of optimal formulations for specific biomedical purposes. In the context of 3D cell culture, this capability could streamline the development of scaffolds for applications ranging from regenerative medicine to the formation of complex tissues such as muscles and tendons.

Teachable Machine appears to be a reliable AI, particularly as it was developed by Google, a well-known and respected company in the field of technology and machine learning [29]. Moreover, it has been utilized by many developers and academics to successfully train and deploy image classification models, as evidenced by numerous tutorials and guides available online [30]. However, like any tool, the accuracy and reliability of predictions made by a model trained using Teachable Machine depend on the quality of the training data and the model’s suitability for the specific task [31].

**Figure 5:**
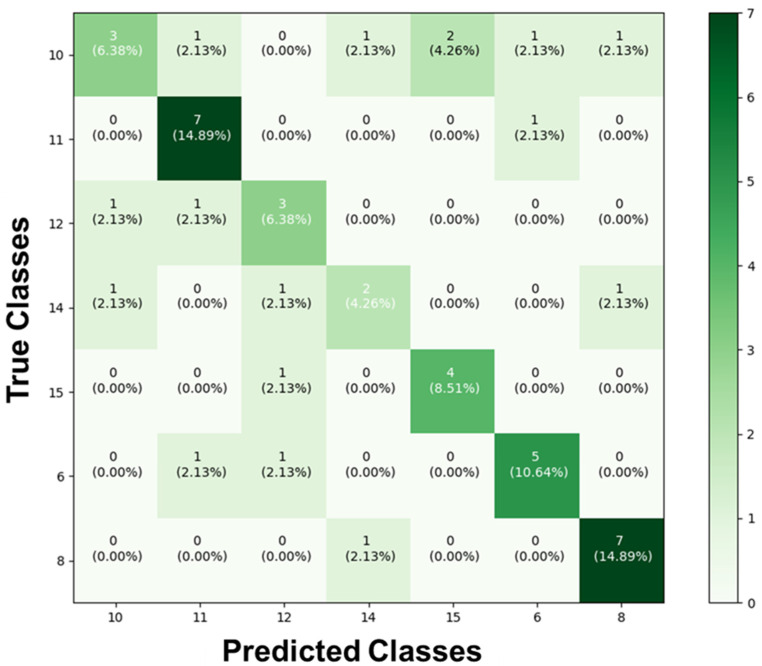
Confusion matrix of the prediction for the seven distinct types of hydrogels. This matrix presents the proportions corresponding to the identifications of the forty-seven images, based on the inputs from the classes DMEM-6, DMEM-8, DMEM-10, DMEM-11, DMEM-12, DMEM-14, and DMEM-15. It reveals a significant disparity in the AI’s prediction classifications.

As for the results of the test to identify the different classes and determine to which specific hydrogel the images of stretched and unstretched filaments belonged, they were collected and added to the confusion matrix. When we compared the obtained results with the actual values of the images in each section, the confusion matrix shown in Figure 5 was constructed. It reveals a significant disparity in the AI’s prediction classifications. Among all the groups presented and trained, substantial accuracy was achieved only for the stretched and unstretched filaments of DMEM-8 (14.89%) and DMEM-11 (14.89%). For the remaining classes, there was confusion during the predictions, where different types of filaments were misclassified in terms of the hydrogel to which they belonged.

Within the field of machine learning statistics, there is a term known as overfitting, which occurs when a statistical model fits precisely to its training data, including noise and random fluctuations in the data. This results in a model that performs well on the training data but poorly on new and unseen data, undermining its purpose [32,33]. This may be one of the reasons explaining the lack of consistency between the images predicted by the AI and their actual values.

The Teachable Machine artificial intelligence has been used in various types of studies and research involving imaging for different applications, such as tympanic membrane differentiation [30], melanoma tomographies [34], and plant pest-related diseases [35], as well as studies focused on children’s education and development [7,36]. Using AI as a tool for this work, the results obtained have been positively surprising due to its effectiveness. Furthermore, it has impressed by demonstrating precise accuracy, proving to be highly capable of distinguishing proportionally between the categories of images F and FE after its training, resulting in consistently high accuracy rates. On the other hand, it was found that the AI’s performance was unsatisfactory in predicting the various classes of filaments based on the type of hydrogel, encompassing both stretched and unstretched ones. This was partly due to the similarities in composition and consequently similarities in the generated images, even though other attributes, such as micro- and nanomechanical properties, were distinct.

The chemical and physical composition of the filaments plays a crucial role in the accuracy of the classifications made by the artificial intelligence, since differences in structural properties, such as color and morphology, can directly influence the distinction between classes, as observed in the classification results of stretched and unstretched filaments. The similarity between some of the attributes of the hydrogels, such as their composition and mechanical properties, may have contributed to the classification difficulties faced by the AI, especially when dealing with images of different types of filaments, such as those of DMEM-8 and DMEM-11. The variation in the results can be explained by the influence of the chemical compounds used and the presence of microscopic and nanomechanical differences in the materials, which directly affect their properties and behavior during the analysis. These characteristics, together with the mechanical properties and degradation over time, have been widely discussed by Araujo Neto, L.A. (2024) [37,38].

To enhance Teachable Machine’s ability to improve image prediction accuracy, the following enhancements can be considered: (i) an increase in the diversity and quantity of training data, representing an even wider range of hydrogel variations, could enhance the model’s capability to identify specific fiber classes [39]; (ii) the incorporation of more advanced image preprocessing and segmentation techniques can assist in highlighting distinctive features in the images, making them more easily recognizable by the algorithm [40]; and (iii) optimizing the parameters of the machine learning model, such as the choice of classification algorithm and neural network architecture, is essential to ensure more accurate and consistent performance [41]. Finally, a more detailed analysis of the model’s limitations and the types of errors it makes can guide specific adjustments to enhance the accuracy of image prediction.

## 4. Conclusions

The images obtained from histological cross-sections revealed that variations in the polymer proportions used in filament manufacturing were responsible for the differences in coloration observed in each. Furthermore, stretched filaments underwent changes in their conformation due to increased material compaction during the stretching process, which distinguished them from unstretched samples. The utilization of machine learning for analyzing images obtained through light microscopy of filament sections showed promising results, albeit with limitations in predicting specific classes. Teachable Machine’s AI demonstrated high accuracy in distinguishing between categories F and FE but encountered difficulties in accurately predicting different fiber classes based on the type of hydrogel. Therefore, to enhance the Teachable Machine’s image analysis capacity, it is crucial to consider diversifying and expanding the training data, implementing advanced image preprocessing and segmentation techniques to highlight distinctive features, and optimizing machine learning model parameters, including the choice of classification algorithm and neural network architecture, to ensure more precise and consistent predictions. Beyond their immediate use in histological studies, the hydrogels analyzed here represent a promising platform for 3D cell culture systems. By leveraging their customizable mechanical and morphological properties, they could support the formation of functional tissues for biomedical applications, including neural networks, tendons, and muscular systems. These findings underscore the broader potential of such materials in advancing tissue engineering and regenerative medicine.

## Figures and Tables

**Figure 3:**
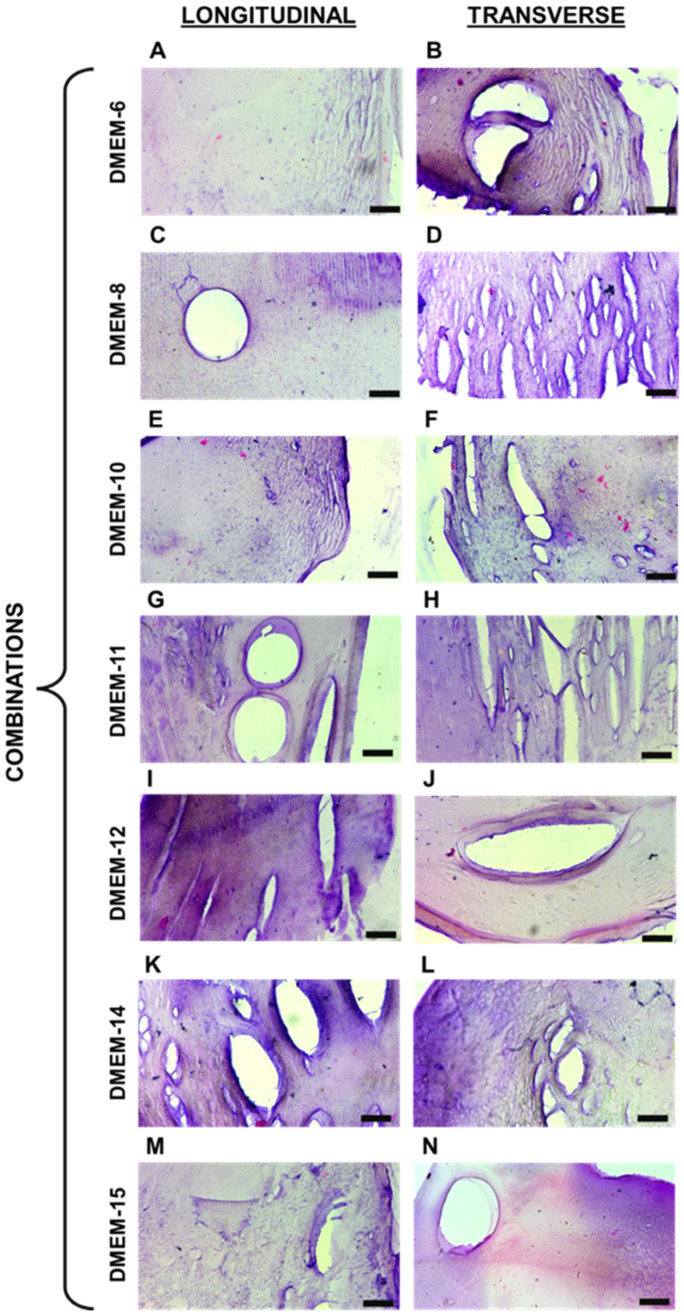
Histological sections of stretched hydrogel filaments: (**A**,**C**,**E**,**G**,**I**,**K**,**M**) representative images of longitudinal sections; (**B**,**D**,**F**,**H**,**J**,**L**,**N**) representative images of transverse sections. The black mark in the bottom right corner of each image represents 100 μm.

**Table 1:** Names for the combinations of independent variables, representing the relative concentrations (% *w*/*v*) of sodium alginate and gelatin in the hydrogels, and calcium chloride solution as the crosslinking agent.

	Polymers		Crosslinker
Combination’s Name	Sodium Alginate (%)	Gelatin (%)	CaCl_2_ (%)
DMEM-6	7	3	3
DMEM-8	7	5	3
DMEM-10	8.364	4	2
DMEM-11	5	2.318	2
DMEM-12	5	5.682	2
DMEM-14	5	4	3.682
DMEM-15	5	4	2

## Data Availability

The data are contained within this article.
